# Transgenic Breeding Approaches for Improving Abiotic Stress Tolerance: Recent Progress and Future Perspectives

**DOI:** 10.3390/ijms21082695

**Published:** 2020-04-13

**Authors:** Ali Anwar, Ju-Kon Kim

**Affiliations:** Graduate School of International Agricultural Technology and Crop Biotechnology Institute/GreenBio Science & Technology, Seoul National University, Pyeongchang 25354, Korea; anwar_ali@snu.ac.kr

**Keywords:** plant biotechnology, transgenic breeding, abiotic stress, CRISPR/Cas9, QTL, miRNA

## Abstract

The recent rapid climate changes and increasing global population have led to an increased incidence of abiotic stress and decreased crop productivity. Environmental stresses, such as temperature, drought, nutrient deficiency, salinity, and heavy metal stresses, are major challenges for agriculture, and they lead to a significant reduction in crop growth and productivity. Abiotic stress is a very complex phenomenon, involving a variety of physiological and biochemical changes in plant cells. Plants exposed to abiotic stress exhibit enhanced levels of ROS (reactive oxygen species), which are highly reactive and toxic and affect the biosynthesis of chlorophyll, photosynthetic capacity, and carbohydrate, protein, lipid, and antioxidant enzyme activities. Transgenic breeding offers a suitable alternative to conventional breeding to achieve plant genetic improvements. Over the last two decades, genetic engineering/transgenic breeding techniques demonstrated remarkable developments in manipulations of the genes for the induction of desired characteristics into transgenic plants. Transgenic approaches provide us with access to identify the candidate genes, miRNAs, and transcription factors (TFs) that are involved in specific plant processes, thus enabling an integrated knowledge of the molecular and physiological mechanisms influencing the plant tolerance and productivity. The accuracy and precision of this phenomenon assures great success in the future of plant improvements. Hence, transgenic breeding has proven to be a promising tool for abiotic stress improvement in crops. This review focuses on the potential and successful applications, recent progress, and future perspectives of transgenic breeding for improving abiotic stress tolerance and productivity in plants.

## 1. Introduction

The world’s population is very intensively increasing every day; it is estimated that it will increase to 9.7 billion by 2050 [1]. The intensive increase in biotic and other environmental stresses (high and low temperature, salinity, drought, and heavy metal stresses) due to climate changes can pose a severe threat to tropical crop production [2]. Although conventional breeding techniques have significantly improved crop production and yields, new methodologies and techniques are needed for crop production improvement to fulfill the food demand [3]. Additionally, environmental stresses are defined as the adverse effects of non-living factors on living organisms (plants) in a specific environment. Abiotic stresses include drought, heat, cold, and heavy metals [2]. Excess of salts or toxic metals, such as aluminum, arsenate, and cadmium, in the soil are major environmental factors that significantly influence the growth of plants and lead to a decline in plant productivity [4].

**Figure 1:**
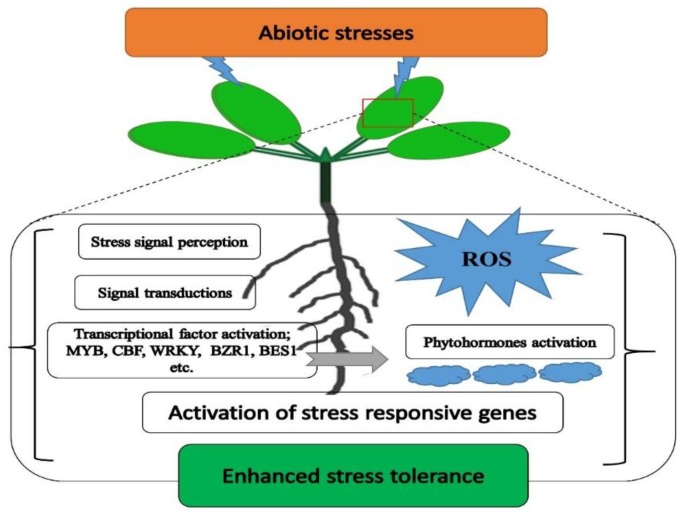
A simplified scheme of mechanism for abiotic stress tolerance in plants. Abiotic stress is involved in the overproduction of reactive oxygen species (ROS), which causes cell death. A complex plant defense system stabilizes the ROS production and protects the plant cells.

To improve plant survival and production efficiency, different approaches have been established to improve plant resistance [3,5]. The exposure of plants to these environmental stresses leads to reactive oxygen species (ROS) overproduction, which negatively affects the activities of enzymes, the biosynthesis of carbohydrates, DNA, proteins, and other biochemical activities, thus leading to oxidative stress (Figure 1) [6,7]. ROS also influence the expression of a number of genes involved in many processes, including growth, the cell cycle, programmed cell death (PCD), abiotic stress responses, pathogen response, systemic signaling, and development [8]. The defense system (superoxide dismutase (SOD), peroxidase (POD), catalase (CAT), glutathione reductase (GR), ascorbate peroxide (APX) enzymes, etc.) plays a key role in the regulation of the production of ROS and hence protects plants from abiotic stresses [4]. 

To meet the growing demand for food and to contrast the harmful effects of abiotic stress on plant production, it is indispensable to produce transgenic lines that have improved resistance to a wide range of abiotic stresses [6,9,10]. The traditional breeding techniques were used for genetic variations arising from varietal germplasms and intergeneric or interspecific hybridizations to induce mutations at a cell and tissue culture level to increase the plant’s resistance to environmental stresses. However, these techniques have many limitations [11]. A long time is required to introduce new plants and numerous undesirable genes can be transferred along with desirable genes, there is a low yield improvement under stress conditions due to the complexity of the stress response and its mechanisms, and there is no guarantee of obtaining a particular gene combination over millions of crosses [12]. 

The recent advances in biotechnology have dramatically changed the capability of gene discovery and functional genomics in plants to regulate a specific character [10,13]. Biotechnology techniques make protein and metabolite profiling easy and allow us to understand the physiologically complex processes and cellular functions [11]. Current efforts to improve the abiotic stress tolerance of plants resulted in significant achievements [14]. Plant biotechnology and genetic engineering approaches for abiotic stress tolerance [15] are based on the gene expression that is involved in regulatory and signaling pathways that control genes encoding stress-resistant proteins and enzymes for the synthesis of functional metabolites [10,16,17,18]. 

## 2. Physiological and Biochemical Mechanisms of Abiotic Stress

Plants’ exposure to abiotic stress leads to diverse physiological and biochemical changes, such as overproduction of reactive oxygen species (ROS: H_2_O_2_, O_2_^−^, OH) [5]. Plants possess a very efficient defense system (including SOD, POD, CAT, GR, antioxidant enzymes, etc.), which is involved in plant protection during stress conditions [8]. The overproduction of ROS influences the expression of numerous genes involved in chlorophylls, photosynthesis, enzymes, cells, and the defense response [2]. The hydrogen peroxide signaling pathway (H_2_O_2_) plays dual roles: at lower concentrations, it activates the plant’s defense system to reduce the harmful effects of abiotic stress, while it is highly toxic at higher concentrations, causing negative effects on the plant’s defense system [4,8]. 

**Figure 2:**
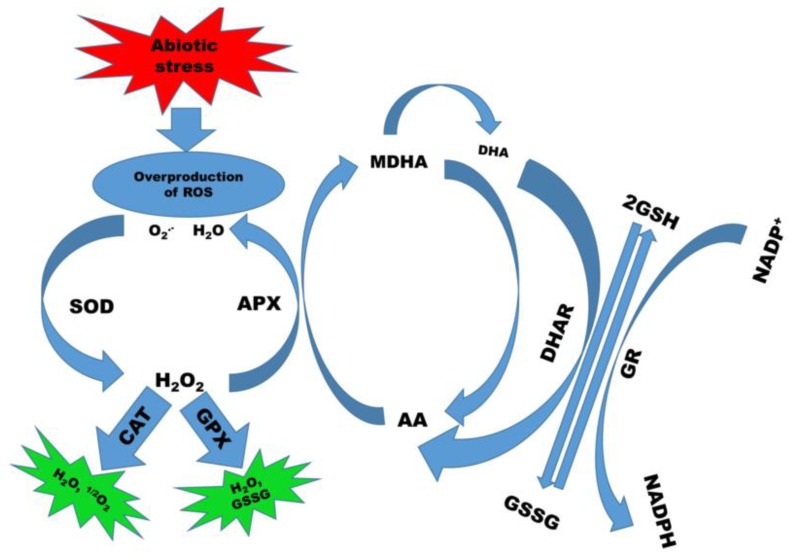
The mechanisms of plant defense systems. Plant antioxidants (superoxide dismutase (SOD), peroxidase (POD), catalase (CAT), glutathione reductase (GR), ascorbate peroxide (APX), etc.) act as the plant defense system, which reduces the toxic effects of abiotic stress, as reported previously [8].

In the past decade, several candidate genes/transcription factors (TFs) have been identified to be involved in plant defense systems. MYB transcription factors have been identified as being involved in a wide range of regulatory networks, including metabolism and biotic and abiotic stresses [19]. Similarly, the *ZmPP2C-A* (*protein phosphatase type 2C clade A*) gene was identified to facilitate drought tolerance in maize [20]. The author reported that PP2C (protein phosphatase type 2C) was involved in ABA biosynthesis and defense-related genes to reduce the harmful effects of drought stress. As displayed in Figure 2, plant defense-related enzymes (SOD, CAT, POD, GR, etc.) are involved in decreasing the overproduction of ROS [8].

## 3. Biotechnology Applied to the Breeding of Abiotic Stress Tolerance

Plant biotechnological approaches, such as molecular breeding and genetic engineering, offer the possibility to obtain improved and genome edited crops in a short time [3,10]. Genetic engineering may also allow us to overcome the reproductive barrier among different plant species [10]. Transgenic breeding efficiently improved crop production through genetic modification and improvements, with a short breeding phase [21]. Genome sequences are now available for different plant species (Arabidopsis, cucumber, tomato, rice, etc.), including species with large and complex genomes [22,23]. Moreover, the advent of so-called ‘next-generation sequencing’ technologies offers the possibility to sequence, relatively quickly and cheaply, new crops and different varieties of the same crop to develop new markers to be used in molecular breeding [24,25].

As improvements in plant physiology enhance our knowledge on the complexity of drought-tolerant mechanisms and its relation to different traits, selection efficiency using molecular and genomic approaches will result in the identification of quantitative trait loci (QTL) and genes linked with traits [26]. The identification of the candidate genes responsible for plant tolerance under different abiotic stresses is essential for developing transgenic crops with enhanced drought stress tolerance [12]. Once the genes controlling drought tolerance have been identified through QTL mapping, these genes can then be incorporated into the genetic background of any desirable cultivar using genetic engineering and hybridization with marker-assisted selection [10,21,23]. 

Plant engineering strategies for abiotic stress tolerance were intensively studied over the last two decades. Scientists investigated the gene expression that was potentially involved in plant defense signaling pathways that encoded proteins conferring abiotic stress tolerance. The current efforts to improve plant stress tolerance through genetic transformation have resulted in several important achievements [10]; however, the genetically complex mechanisms of abiotic stress tolerance make the task extremely difficult [3]. Therefore, plant biotechnology should be fully combined with conventional breeding and physiology. The molecular mechanisms of abiotic stress tolerance, the number of stress responsive genes, the transcription factors, and the signaling pathways are reported in several extensive reviews and research that was recently published. 

## 4. Quantitative Trait Loci (QTL)

Quantitative trait loci (QTL) mapping is an emerging technique in plant breeding [26] and is a perfectly adequate method for investigating genetically complex systems for the tolerance of abiotic stresses [27]. The enhancement of crop production under environmental/abiotic stress conditions was achieved through QTL manipulation, which controlled specific agronomic traits and physiological mechanisms to enhance crop production. Quantitative trait loci can be categorized as “adaptive and constitutive” according to their stability and response to abiotic stresses [28]. The constitutive QTL are frequently discovered in most environments, but the adaptive QTL are detected under specific environmental conditions, such as QTL that are expressed under increasing or decreasing temperature, thus indicating that QTL are responsible for controlling temperature stresses [27]. 

Studies identified specific resistance loci through QTL mapping and assessed the race-specificity of resistance and interactive genes involved in the plant developmental process under specific environmental conditions [26,29]. These studies provide insights into the number of QTL involved in complex disease resistance, epistatic and environmental interactions, race-specificity of partial resistance loci, interactions between pathogen biology, plant development and biochemistry, and the relationship between qualitative and quantitative loci [30]. QTL mapping also provides a framework for the marker-assisted selection of complex disease resistance characters and the positional cloning of partial resistance genes [29]. 

A number of important QTL were identified to control key traits in plants. Recently, 25 QTL were identified for timing of flowering (TOF), 17 QTL for spring yield, and 6 QTL for cumulative summer biomass in *Medicago sativa* L [30]. Three TOF-related QTL were stable, and four TOF-QTL were detected in the corresponding genomic locations of the flowering QTL of *M. truncatula*, an indication of possible evolutionarily conserved regions [29]. The potential candidate genes for the SNP sequences of QTL regions were identified for all three traits, and these genes would be potential targets for further molecular studies [30]. 

These QTL, markers and the potential candidate genes associated with spring flowering time and the biomass yield of alfalfa constitute valuable genomic resources for improving these traits via marker-assisted selection (MAS). Another study reported five major QTL, *qHII-1-1*, *qHII-1-2*, *qHII-1-3*, *qHII-2-1*, and *qCC-1-5* (*qREC-1-3*), in tomatoes involved in high temperature stress tolerance [30]. The authors reported that *qHII-1-1*, *qHII-1-2*, and *qHII-1-3* were located, respectively, in the intervals of 1.43, 1.17, and 1.19 Mb on chromosome 1, while *qHII-2-1* was located in the interval of 1.87 Mb on chromosome 2. 

**Figure 3:**
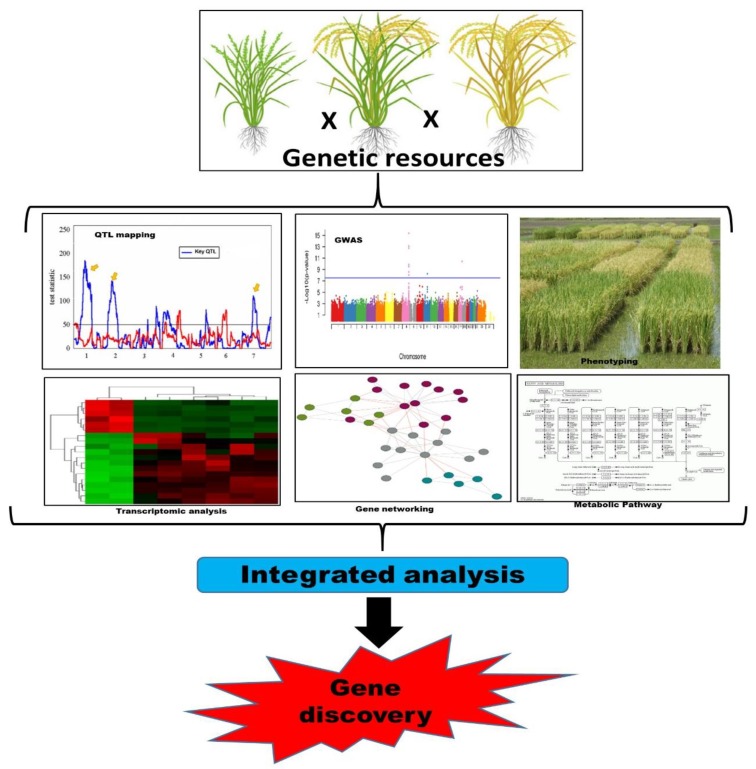
A schematic model for quantitative trait loci (QTL) mapping and the discovery of functional genes for a specific trait. To discover candidate genes for specific traits, a number of plant populations were used as a genetic resource. These resources were QTL mapping, GWAS (genome-wide association study), and transcriptomic analysis to allocate candidate genes for control of a specific character.

The locations observed with conventional QTL mapping and QTL-seq were consistent. *qCC-1-5* and *qREC-1-3* for CC (chlorophyll contents) and REC (relative electrical conductivity), respectively, were located at the same position observed by conventional QTL mapping [31]. These findings were also confirmed by RNA-seq, and four candidate genes (*SlCathB2, SlGST, SlUBC5,* and *SlARG1*) associated with heat tolerance were finally detected within the major QTL by DEG analysis, qRT-PCR screening and biological function analysis. The findings concluded that the combination of conventional QTL mapping, QTL-seq, and RNA-seq analysis can rapidly identify candidate genes, thus greatly shortening the breeding process and improving the breeding efficiency [31]. QTL can work as an excellent tool for translational genomics and the breeding of quantitative traits in a variety of plants through comparative genomics approaches [26,27,29,30]. These studies clearly demonstrated the importance of QTL identification and hence offered new and unique markers (Figure 3) with great capabilities to contribute to and improve abiotic stress tolerance and yields.

## 5. miRNAs in Abiotic Stress Tolerance

MicroRNAs (miRNA) are a class of single-stand RNA molecules that are 21 to 24 nucleotides in length [25]. Plant miRNAs are frequently investigated as key regulators of specific gene expression during the plant developmental process, mediating a defense response against biotic and abiotic stresses [32]. A phosphate group is attached at the 5′ end and a hydroxyl group at the 3′ end of mature miRNA. miRNA targets the 3′ UTR region of specific mRNA, thereby provoking the silencing of post-transcription, causing the degradation of mRNA and/or inhibition of its translation [33,34,35]. In the past decade, researchers mainly aimed to identify miRNAs with a great capability to induce abiotic (chilling, drought, and heavy metal) stress tolerance. A previous study found miRNAs that are involved in a wide variety of processes in plant cells, including development, transcription, protein degradation, and detoxification (Table 1) [36,37,38]. 

**Figure 4:**
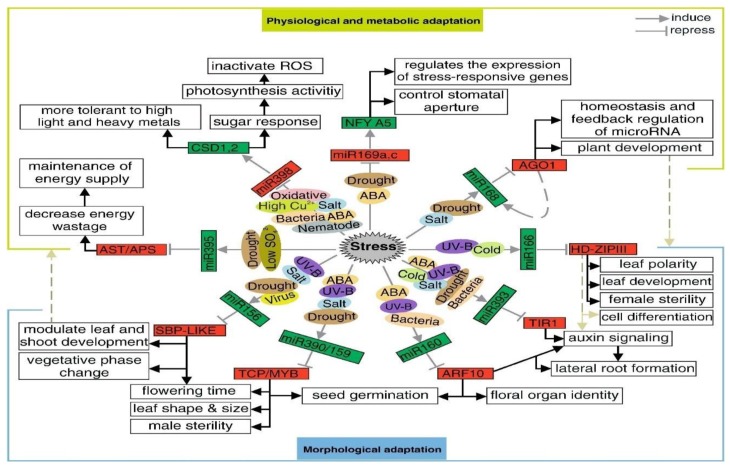
The regulatory mechanisms of miRNAs under abiotic stress conditions in Arabidopsis [32]. The proposed network defines the molecular mechanism of miRNA response to a variety of biotic and abiotic stresses. The network is based on expression profiles and targeted gene transcripts under stress condition.

Here, we discuss a current overview of miRNA identification, functions, specific roles, and the mechanisms in major crops for abiotic stress tolerance, as well as the recent case studies for improving abiotic stress tolerance through overexpressing selected miRNAs (Figure 4) [37]. In *brassica napus,* farnesyltransferase (alpha-subunit) confers resistance to seed abortion during flowering, which is thought to be influenced by water deficiency during drought stress [38]. miR393 was observed to be strongly up-regulated by dehydration, cold, high salinity, and ABA treatments in various plant species [36,37]. In Arabidopsis, miR169 was down-regulated by drought stress, and its target nuclear factor YA5 (NF-YA5) was significantly induced upon exposure to drought stress [34,39]. The miRNA169a-overexpressing plants were enhanced by leaf water loss and were highly sensitive to drought stress, when compared to wild type plants, while the miRNA169a-targeted NF-YA5-overexpression increased drought stress tolerance [40]. 

Likewise, the characterized *GmNF-YA3* gene, a target of miR169, led to significantly improved tolerance of drought stress in *Arabidopsis thaliana* (Figure 4) [41]. The researchers also reported that the over-expression of GmNF-YA3 in Arabidopsis resulted in increased sensitivity to salinity and ABA stresses [40]. Tomato miR169 overexpression lines induced drought stress through its four targets NF-YA1/2/3 and multidrug resistance-associated protein gene 1 (*MRP1*), which were all down-regulated [42]. Constitutive over-expression of tomato miR169c improved drought stress tolerance as compared to wild-type plants through reduction of leaf water loss, the stomatal opening, and transpiration rate [42]. The *Agrostis stolonifera* plants overexpressing a miR319 rice gene *Osa-miR319a*, increased their wax contents in leaves for the retention of water and reduced their Na^+^ ion uptake. Thus, they induced salinity and drought stress tolerance [43]. 

Gene expression analysis indicated that the enhanced abiotic stress tolerance can be attributed to a significant down-regulation of at least four putative turf miR319 target genes, teosinte branched/cycloidea/proliferating factors (TCP) *AsPCF5*, *AsPCF6*, *AsPCF8*, *AsTCP14*, and a homolog of a rice NAC (NAM, ATAF, and CUC) domain gene *AsNAC6*0 [42,43,44]. Additionally, overexpression of *Osa-miR319* enhanced leaf size, enlarged stem size, and reduced the number of tillers of *Agrostis stolonifera* [43]. Numerous conserved miRNAs have been identified in *Medicage trunctula* plants under water-deficit conditions [45]. Among these, miR169 was down-regulated in the roots, while miR408 and miR398a/b were highly expressed in the shoots as well as the roots. The miR171 family induced drought stress tolerance in potatoes [46].

The overexpression of the *Osa-miR319* gene led to increased cold stress tolerance (4 °C) after chilling acclimation (12 °C) of plants as compared to wild-type plants [43,47,48]. The miR319 overexpression lines showed more tolerance to cold stress than the *OsPCF5* and *OsTCP21* RNAi lines in rice [49]. miR398 is involved in plant thermo-tolerance mechanisms, especially for the protection of the reproductive organs. Researchers reported that miR398 down-regulated its target CSD (copper/zinc superoxide dismutase) genes [50], *CSD1* and *CSD2,* as well as *CCS*, a gene encoding a copper chaperone for both *CSD1* and *CSD2* [32]. The *csd1*, *csd2*, and *ccs* mutants displayed a higher heat stress tolerance than the wild-type plants associated with the increased accumulation of heat stress transcription factors and heat shock proteins and reduced damage to flowers [50]. Thus, it can be concluded that miR398 and its target gene can be useful for crop improvements to enhance heat stress tolerance. 

The overexpression of miR398-resistance from *CSD2* is involved in the enhanced tolerance of various kinds of abiotic stresses [51]. The investigation of interactions between miR398 and its targeted *CSD1* and *CSD2* genes provided a possible description to previous studies [33,34,45]. The transgenic lines of SOD with miR398 targeted sites negatively impacted the miR398-mediated gene regulation. Based on the abovementioned facts, we concluded that miRNAs played a fundamental role in the improvements of abiotic stress.

## 6. The Development of Abiotic Stress-Tolerant Crops by CRISPR (Lustered Regularly Interspaced Short Palindromic Repeats)/Cas9

**Figure 5:**
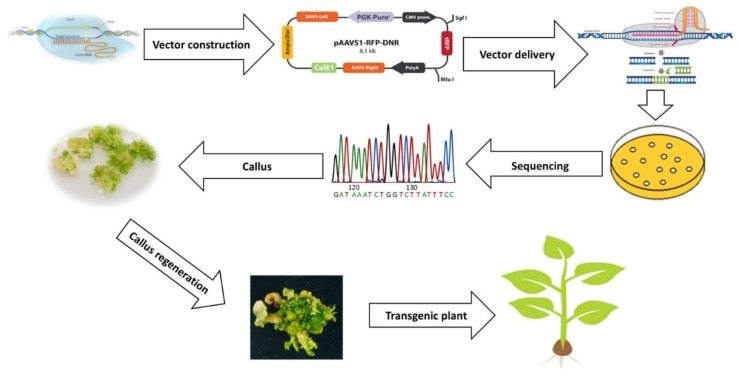
Overview of plant genome editing using the CRISPR (lustered Regularly Interspaced Short Palindromic Repeats)-Cas9 system.

Plants are challenged by different kinds of abiotic stresses, such as salinity, drought, temperature, and heavy metals, which significantly limit crop production around the world [6]. Conventional breeding techniques have been used for crop improvements, which are now becoming constrained by the declining genetic resources of plants and time consuming [21]. There is a crucial need for efficient crop improvement strategies with novel genome editing techniques, such as CRISPR-Cas9 [69], which is easy, fast, efficient, and accurate in obtaining new genome edited crops (Figure 5). Global warming is leading to increases in several kinds of abiotic stress, including temperature and drought, which are major abiotic stresses that limit crop production [70]. Scientists are screening to determine candidate genes using CRISPR-Cas9 technology to overcome these environmental problems.

The *KUP* (K^+^ uptake permease) gene family is the largest family of K^+^ transporters in Arabidopsis [71]. Recently, researchers discovered that differences in cassava genotypes influenced drought resistance through the differential expression of *KUP* genes [72]. A genome-wide study revealed that *MAPKKK* (mitogen-activated protein kinase kinase kinase) genes played important roles in the tissue development of cassava and its resistance to drought stress [73]. When considering tropical climates, *Elaeis guineensis* is the main source of edible oil in Africa and is very sensitive to low temperature, but resists drought and salinity well [23]. *WRKY* genes demonstrated tissue-specific expression under low temperature stress in oil palms [70].

Almost all the *EgWRKY* genes were upregulated under abiotic stresses [74], which suggested that the expression of *EgWRKY* genes in African oil palms play an important role in the responses to abiotic stresses [49]. The CRISPR/Cas9 technology has not been fully applied for crop improvements; thus, the opportunity exists to apply the technology for a variety of crop genetic modifications for enhanced yield and biotic/abiotic stress tolerance.

Recently, a plant multi-genome editing toolkit was developed using a CRISPR/Cas9 binary vector set and a gRNA module vector set [23,69,75]. This will make it easier to use CRISPR/Cas9 in a variety of plant systems and is especially useful for high-efficiency multiplex plant genome editing. Therefore, the direct introduction of cas9 and sgRNA into host cells by genetic transformation is the only requirement for plant genome editing with regard to abiotic stresses [76]. The previous study demonstrated that gemini virus replicons (gvrs) could be used to transfer cas9/sgRNA to plant cells with enhanced mutations when the replication initiation protein gene (rep) was co-transformed with Cas9/sgRNA structures [71]. In addition, to develop the practicability of CRISPR-cas9 technology, more efforts are required to make targeted genome editing in plants easier and faster [7,71].

Two recent reports using direct delivery of the tobacco rattlesnake virus (Trv) [75] and cabbage leaf virus (Calcv) [77] clearly demonstrated the feasibility of different virus-mediated Cas9/sgRNA delivery for efficient plant genome editing. CRISPRi (CRISPR interference) in plants was proven to regulate the transcription of target genes in plants stably and effectively under the guidance of RNA by fusing inactivated dCas9 into the effect domain [76,78]. dCas9 was used in functional genetics to regulate gene expression and new synthetic biological applications [79]. gRNAs were used to recruit them into specific DNA sequences as fusion proteins with transcription factor activation or inhibition domains [80,81].

The transcription of the reported constructs and endogenous *PDS* (*phytoene desaturase*) genes in tobacco [81] was regulated by fusing dCas9 C terminal into the EDLL domain as a transcriptional activator and into the SRDX domain as a repressor. The DCAS9/SGRNA/effector recognition complex interfered with effective SGRNA-dependent induction and the reversible suppression of gene expression transcriptional regulation [82,83]. The target site of effective CRISPRi should be between -50 BP and +300 BP relative to the transcription initiation site (TSS). The CRISPR activator (crispr a) system regulates the gene expression in the range of 1000 times the expression of a single sgRNA at a binding site [84]. The development of genome-scale crispri and crispra libraries will provide significant tools for the functional genomic study of stress response signaling pathways [85]. The availability of online resources for CRISPR/Cas system materials and tools ensures that the wide adoption and application of this technology remains very simple. This includes web resources for the CRISPR/CAS system and software tools of sgRNA design.

## 7. Transgenic Breeding to Improve Abiotic Stress Tolerance

### 7.1. Drought Stress

Drought is a key threat for plant production around the world, due to insufficient rainfall or a lack of availability of irrigation water [86]. Globally, one-third of total agricultural land is arid or semi-arid, due to insufficient water [87]. Thus, drought stress, together with other climatic changes, causes a significant loss of crop yield [88]. According to previous reports, the global temperature increased by 1.2 °C in the last century and was expected to have increased an additional 3 °C by 2010. The development of crop plants with improved performance under drought stress is therefore a major breeding objective for scientists. Transgenic breeding or biotechnology approaches play a fundamental role in mining candidate genes potentially involved in drought stress tolerance [3,10]. Various molecular techniques (marker-assisted selection (MAS) and genomic selection (GS) and QTL-mapping are used to identify candidate gene (that’s can control a specific character), and CRISPR-Cas9 is being used to make transgenic lines for the drought stress tolerance of crops (Supplementary Table S1) [34].

Plant hormones are involved in the response to drought stress, and they play a crucial role in drought stress tolerance by regulating multiple processes, such as stomatal closure, root growth, and the production of protective metabolites [5]. In the model plant Arabidopsis, *sucrose non-ferment 1 related kinase 2 protein kinases* (subgroup III SnRK2) are reported to function as serine/threonine protein kinases and as central and positive regulators of the ABA signaling pathway [88]. Upon the perception of ABA, the ABA receptor PYR (pyrabactin resistance 1)/PYL (PYR1-like protein)/RCAR (regulatory components of the ABA receptor) proteins inhibit the activity of clade A PP2C phosphatases, thus releasing the *SnRK2s* to phosphorylate the downstream proteins [89].

Under drought stress, *SnRK2s* phosphorylate (especially RK2.6) the key ion channel *KAT1* (*inward rectifying potassium channel 1*) and *SLAC1* (guard call slow ion channel 1) to facilitate stomatal opening [90]. The activated *SnRK2* can also phosphorylate AREB/ABFs (ABA responsive protein) and bZIP transcription factors and upregulate the expression ABA responsive genes, thus activating the ABA signaling pathway to induce drought stress tolerance [91]. The ABA independent transcription factor (DREB1A) transgenic plants improve water use efficiency compared to wild type plants [84].

DREB1A distinctly stimulated the response of groundnut roots when exposed to water scarcity, wherein the stimulated roots grew significantly longer, especially in the deeper layers of soil [21]. Transgenic DREB1A groundnut (DREB1A genetically transformed by *rd29* promoter), led to altering the root system in a consistent manner throughout the soil profile, thus increasing the root length density to facilitate the water extraction frequency [92]. Thereby, the transgenic groundnut resulted in enhanced pod yield, enhanced yield components, and enhanced harvest index.

Most drought inducible genes are activated by drought responsive transcription factors (TF), such as NAC, MYB, etc., which regulate drought stress tolerance [93,94]. NAC is a domain name derived from the first letters of three different genes, including NAM (no apical meristem), ATAF (Arabidopsis transcription activation factor), and CUC (cup-shaped cotyledon), which are involved in plant developmental and abiotic stress responses [95,96,97,98]. A previous study reported that *OsNAC6* mediated root structural adaptations, enhanced the number and diameters of roots, and increased drought stress tolerance in rice [87].

**Figure 6:**
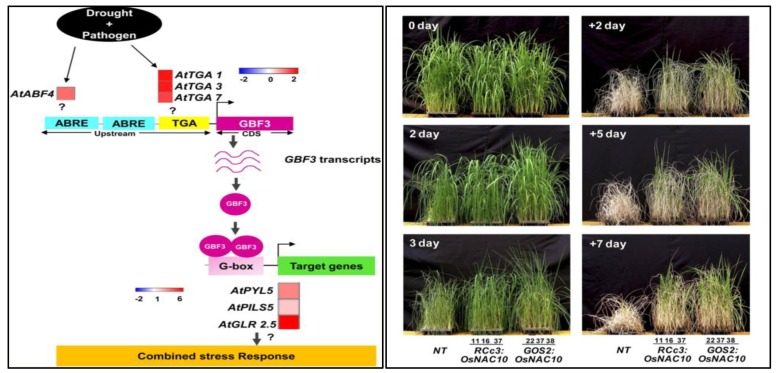
The regulatory mechanism of GBF3 in a combined drought and Pseudomonas syringae stress tolerance [105]. Root-Specific expression of OsNAC10 increased drought stress tolerance and grain yield in rice [96].

Moreover, *OsNAC10* is another member of the NAC family, involved in drought stress tolerance in field conditions and also increases the grain yield in rice, as presented in Figure 6 [96,99]. Similarly, the overexpression of *OsNAC5* enlarges the root diameter in rice and leads to increased drought stress tolerance and grain yield in the field [99,100]. The overexpression of *AtNAC2* demonstrated an improved tolerance under limited water conditions, providing a candidate gene for water stress tolerance in crops [101,102,103]. The overexpressed *MuNAC4* gene performed a substantial part in successful water stress tolerance by reducing any injury to membrane structures and enhancing the osmotic and antioxidative enzyme regulation in horse gram [104].

The *G-Box Binding Factor 3* (*GBF3*) TF (transcription factor) gene reported in *Arabidopsis thaliana* showed a significant resistance to drought and heat stress. *AtGBF3* is a member of the basic leucine zipper (bZIP) superfamily and binds to the palindromic, hexameric “G-box” element (CACGTG) in the promoters of many stress-responsive genes [105]. The *AtGBF3* protein comprises a proline-rich domain at the N-terminal and a basic leucine zipper domain at the C-terminal, conferring tolerance to individuals and combined drought and pathogen stress responses [106]. *AtGBF3*-overexpressing plants were tolerant under individual and combined drought and *Pseudomonas syringae* pv. tomato infection stresses, when compared to control plants, as presented in Figure 6. The global transcriptome of *Atgbf3* mutants under combined stresses led to the identification of its downstream targets, *PYL*, *PIL*, and *GLR* [92]. Researchers confirmed that *AtGBF3* regulated the combined stress tolerance by activating ABA-mediated signaling. They concluded that *AtGBF3* significantly regulated combined drought and *Pseudomonas syringae* stresses [21,105].

MicroRNAs played important roles in plant responses to environmental stresses, including many developmental processes and yields [26,38]. Plants respond to drought stress by up-regulating or down-regulating the expression of certain miRNAs or synthesizing new miRNAs [36,107]. Some drought stress response miRNAs have been reported in numerous plants, such as *A. thaliana*, *Oryza sativa*, barley, wheat, and soybeans, using high-throughput sequencing technology [34,35,36]. For example, the miR169 family is a conserved and large-scale miRNA gene family in plants, which is involved in abiotic stress tolerance [40].

To date, about 400 miR169 gene family members have been identified in 35 plant species, including monocots, dicots, and some ancient gymnosperms [40,41,107]. The miR169 family in Arabidopsis, rice, and *Populus trichocarpa* consists of 14, 18, and 33 members, respectively. Previous studies have shown that miR169 targets are usually *NF-YA* (*Nuclear Factor Y, subunit A*) family members, widely involved in abiotic stress responses, such as drought, high salt, extreme temperature, and N deficiency, in different plant species [98,108]. miR169 exerts a negative regulatory role in the response to drought stress by inhibiting the expression of its target gene, *nuclear factor YA* (*NF-YA*) [35,40,41].

**Figure 7:**
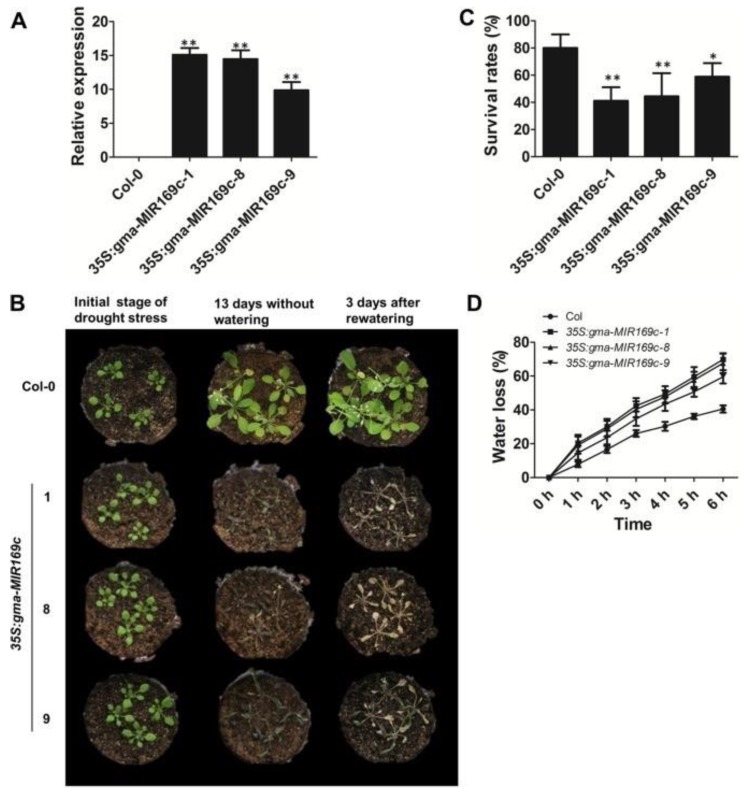
Arabidopsis plants overexpressing the 35S:gma-MIR169c gene show an increased sensitivity to drought stress compared to the wild type * *p* < 0.05;** *p* < 0.01 [35].

Histochemical ß-glucuronidase (GUS) staining showed that the gma-miR169c promoter drives GUS reporter gene expression in various transgenic Arabidopsis tissues, and the stress-induced pattern was confirmed in transgenic Arabidopsis and transgenic soybean hairy roots [35]. Arabidopsis overexpressing *gma-miR169c* is more sensitive to drought stress, with reduced survival, accelerated leaf water loss and shorter root lengths than wild-type plants, as shown in Figure 7. Moreover, researchers identified a precise cleavage site for 10 gma-miR169c targets and found reduced transcript levels of the *AtNF-YA1* and *AtNF-YA5* transcription factors in *gma-miR169c*-overexpressing Arabidopsis and reduced expression of the stress response genes, *AtRD29A*, *AtRD22*, *AtGSTU25*, and *AtCOR15A* [34,35]. These results indicated that *gma-miR169c* played a negative regulatory role in drought stress and is a candidate miRNA for improving plant drought adaptation.

### 7.2. Salinity Stress

Salinity is one of the most serious factors limiting the productivity of agricultural crops. Saline soil has a high concentration of soluble salts [2]. Salinity affected soils contain excessive soluble salts and exchangeable sodium on the surface, which affects plant root systems. According to the FAOs (Food and Agriculture Organization) 2008 report, approximately 800 million hectares of land around the world are affected by salinity [109]. High salinity affects plants in several ways: the alteration of metabolic processes, membrane disorders, irregular cell division and expansion, decreased photosynthetic activity and protein synthesis, increased ion toxicity, and enzymatic disorders [110].

Plants under salinity stress uptake excess amounts of Na^+^ and Cl^−^ ions, thus increasing their accumulation in different tissues of the plants, leading to oxidative stress [8]. However, the accumulation of these ions in plant tissues may also have direct toxic effects by inhibiting protein synthesis, photosynthesis, and susceptible enzymes [8]. Plants respond to salt stress through a series of mechanisms, such as Na^+^/K^+^ homeostasis and Na^+^ exclusion. Additionally, the excess amount of these Na^+^ and Cl^−^ ions in plant cells leads to the overproduction of ROS, which is highly toxic and causes oxidative stress [103,110].

Salt/salinity stress caused numerous physiological and biochemical changes in plants, including osmatic stress, ionic imbalances, and secondary stress [111]. High salinity disturbs the osmatic balance, causing biochemical and enzymatic variation, thus leading to a significant reduction in water and nutrient uptake [79,102]. Researchers suggested that salinity can disturb the overall plant growth by influencing the complex interactions in nutrient uptake and accumulation, hormonal imbalance, and oxidative stress (Supplementary Table S2).

Generally, glycophytes are salt-sensitive plants, and they showed resistance through accumulating osmoprotectants; however, halophytes can survive and grow well under salinity stress (about 200 mM NaCl) [112,113] by employing specific mechanisms to reduce the uptake and accumulation of Na^+^ and Cl^-^ ions, thus reducing the harmful effects [114]. Some species of halophytes have specialized glands to excrete Na^+^ and Cl^-^ ions at the leaves surfaces, while in the case of plant regulation of Na^+^ ions out of the cell, some cells have large vacuoles that can act as a sink for the accumulation of excess Na^+^ through transport into vacuoles [113,115].

During the last two decades, researchers searched for the genes responsible for salinity tolerance. Using biotechnological tools, numerous genes, transcription factors, and miRNAs were identified with effects on salinity stress. For example, *high-affinity potassium transporter* (*HKT*) genes, belonging to the Trk/Ktr (K^+^ transporter)/HKT transporter family, are known to be responsible for regulating the transportation of Na^+^ and K^+^ in higher plants [116]. The first plant *HKT* gene was found in wheat, *TaHKT2;1* [117]. A number of studies reported that *HKT* genes should be involved in the exclusion of Na^+^ from leaves in crops [118,119,120].

SKC1 is a key QTL (encode *OsHKT1;5*) that controls salt tolerance in *Oryza sativa* [121,122]. The previous study presented that Nax1 and Nax2 loci can carry *TmHKT1;4-A2* and *TmHKT1;5-A* (sodium transporter) and have great capability to restrict the Na^+^ accumulation in wheat leaf tissues, thus leading to enhanced salt tolerance [123,124]. Previous studies reported that Na^+^/K^+^ transport is mainly controlled by *HvHKT1* and *HvHKT2* in barley and *SbHKT1;4* in *Sorghum bicolor* and concluded that HKTs significantly control salt tolerance [125,126].

Knockout plants demonstrated more salt sensitivity, and the sodium ion uptake and accumulation in shoots suggested that *OsHKT1;1* is potentially involved in recovering Na^+^ from the leaf blade. It is expressed in the vascular tissues of roots and leaves [127], but its exact role in salt tolerance is unknown. *OsHKT1;4* is mainly expressed in the leaf sheath and encodes a plasma membrane-localized protein [128]. Thus, the *HKT* genes family has great potential in Na^+^/K^+^ homeostasis and is predicted to show broad importance in salinity tolerance. Recently, another study also explored the role of the *HKT* gene in salt tolerance. The researchers reported that the overexpression of *zmKHT1;5* enhanced salt tolerance by activating antioxidant enzyme activities (SOD, POD, and CAT) and reduced the accumulation of MDA and H_2_O_2_ [121,122,123].

**Figure 8:**
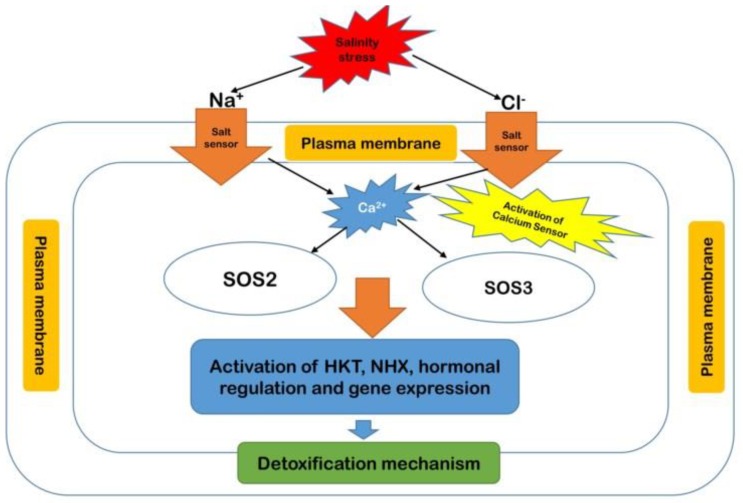
The proposed model of the salt overly sensitive (SOS) pathway for salinity stress tolerance in plants.

The salt overly sensitive (SOS) signaling pathway plays an important role in ion homeostasis and salinity stress tolerance [129]. It consists of SOS1, 2, and 3, and SOS1 is particularly important for Na^+^ and H^+^ transport in the plasma membrane [112]. Moreover, phosphorylation of the SOS3 and SOS2 complexes leads to the activation of SOS1. As mentioned in Figure 8, the newly activated pathway of SOS3-SOS2 stimulates the transcription of SOS1 and stabilizes the cellular levels of SOS1 mRNA [130,131,132].

The phosphorylated form of *SOS1* plays an essential role in Na^+^ efflux and contributes to the reduction of Na^+^ toxicity. Overall, *SOS1* functions as an Na^+^ transporter and mainly exists in the cytosolic compartment of the cell along with the Na^+^ sensor. Previous studies reported that *OsSOS1* of *Oryza sativa* was isolated based on the homology with *AtSOS1* of Arabidopsis [114,133]. Similar to *AtSOS1*, *OsSOS1* encodes a plasma membrane-localized Na^+^/H^+^ antiporter, which mediates the active Na^+^ extrusion in roots under salt stress [133].

The *OsSOS1* knockout line’s response to salt stress is unknown; however, *OsSOS1* had a great ability to resist the salinity stress of the atsos1 mutant, which suggested that OsSOS1 can also play an important role in the salt tolerance of rice [111]. In rice, *OsCIPK24* (*CBL interacting protein kinases*) and *OsCBL4* facilitated *OsSOS1* transport activity to reduce the Na^+^ ion accumulation in the cell and reduce the harmful effects of salinity stress [130] (Supplementary Table S2). The SOS signaling pathway played an important role in salinity stress tolerance in mono- and dicot plants. Thus, we concluded that plant biotechnology plays an important role in identifying candidate genes that are potentially involved in salinity stress tolerance.

### 7.3. Temperature Stresses

Among various environmental factors, “temperature” is considered to be a vital factor that facilitates the ecological distribution of plant species and productivity in various parts of the world. Extreme heat and cold temperatures have a hostile influence on all phases of plant development, growth, reproduction, and yield. Within the plant life cycle, the reproductive stage is the most sensitive to high or low temperature stresses. As reported previously, a very small change in temperature stress (mostly at the flowering stage) can influence a huge amount of yield loss [5].

According to the Intergovernmental Panel on Climate Change (IPCC), plant growth will be challenged with warmer environments as the average surface temperature will increase 2.0–4.5 °C by the end of this century [134]. A previous study reported that changes of 1 °C in temperature will cause great influences on plant physiological and biochemical activities [2]. As temperature stresses become more frequent, there is an urgent need to identify the genes associated with tolerance to temperature stresses and understand their regulatory mechanisms in order to develop crops with enhanced temperature stress tolerance through genetic manipulation (Supplementary Table S3) [134].

Here, we will describe some examples of transgenic breeding for temperature stress. Genetic engineering is defined here as using genes encoding temperature tolerance proteins and metabolites in different crops [135,136,137]. The *omega-3 fatty acid desaturase* gene (*FAD7*) induces chilling stress tolerance in tobacco, due to a strong correlation with the cold responsive gene (*COR*) [138,139].

A number of candidate genes involved in low temperature adaptation are regulated by C-repeat binding factor/dehydration-responsive element binding (*CBF/DREB1*) transcription factors [140,141,142]. Genes encoding a family of cold-regulated (COR) proteins were used to identify a family of Arabidopsis transcription factors known as either *C-repeat binding factors* (*CBF*) (*CBF1*, *2*, and *3*) or dehydration-responsive element binding factors (DREB) (*DREB1B*, *DREB1C*, and *DREB1A*) [142,143]. Three *CBF/DREB1* genes (*CBF3/DREB1a*, *CBF1/DREB1b*, and *CBF2/DREB1c*) belonging to the AP2/DREBP family of DNA-binding proteins were identified in Arabidopsis [144].

In various plants, *CBF1/DREB1b* and *CBF1/DREB1b* overexpression lines increased the chilling stress tolerance, including increased *COR* gene expression levels and proline and sugar accumulation. The overexpression of *CBF1/DREB1b* in Arabidopsis, activated *COR* homologous genes at non-acclimating temperatures [145]. In another study, the Arabidopsis and rice CBF/DREB1-dependent cold response pathway was shown to play a predominant role in the freezing tolerance through the process of cold acclimation [144]. These transcription factors activated the C-repeat (CRT)/dehydration responsive element (DRE) binding factor (*CBF/DREB1*) TFs, which are the key facilitator of *COR* and hence induce chilling stress tolerance [146].

Thus, the *CBF/DREB1* genes are thought to be activators that integrate several components of the cold acclimation response, by which plants increase their tolerance to low temperatures after exposure to nonfreezing conditions [144]. NAC transcriptional factors belong to a large family. Currently, approximately 150 members of NAC have been reported in rice, and they play a crucial role in the abiotic stress tolerance in the plants [100]. In a recent study, researchers reported the characterization of a rice stress-responsive *ONAC095* gene for drought and cold stress tolerance [130]. The overexpression of *ONAC095* (*ONAC095-OE*) and the dominant chimeric repressor-mediated suppression of *ONAC095* (*ONAC095-SRDX*) plants showed comparable phenotypes to wild-type plants under drought and cold stress conditions [147]. These findings indicate that *ONAC095* plays opposing roles in low temperature and drought stress and acts as a positive regulator for low temperature and a negative regulator for drought stress in *Oryza sativa*.

As mentioned above, miRNAs play essential roles in growth, development, and the responses to environmental stress. Many cold stress-responsive miRNAs, including miR396, miR397, and miR319, have been identified in various plant species, such as wheat, rice, Arabidopsis, and tomatoes. Recent studies demonstrated that miR396b positively regulated cold tolerance by repressing ethylene synthesis through reducing 1-aminocyclopropane-1carboxylic acid oxidase (ACO) transcript levels in Arabidopsis [148,149].

The overexpression of miR319 in rice led to enhanced cold tolerance likely via reducing the expression level of the *OsPCF5* and *OsPCF8*, two TCP family transcription factors [134]. Similarly, the overexpression of miR397 significantly improved plant tolerances to chilling and freezing stresses in Arabidopsis by enhancing the expression of cold-regulated C-repeat binding factors (CBFs) and the related downstream genes [33]. In brief, the role of miRNA has been well established as performing a vital role in the cold stress response and adaptation.

Similarly, high temperature or heat stress is also a key barrier for crop production. The exposure of Arabidopsis plants to warm temperatures caused negative effects on the physiological and biochemical activities and growth [2]. During the last two decades, data from genetic engineering on high temperature stress has been accumulating and includes several putative heat sensors, HSF and HSP (heat shock factors and proteins) response pathways and the network of phytohormones, chaperones, and secondary metabolites [132].

A recent study explored the central role of the basic helix–loop–helix (bHLH) transcription factor *PHYTOCHROME INTERACTING FACTOR 4* (*PIF4*) in warmth-mediated morphological acclimation and the acceleration of flowering [150]. Additionally, warm temperatures promoted auxin accumulation and activated the gibberellin (GA) and brassinosteroids (BRs) pathway resulting in hypocotyl elongation [151]. *PIF4* played a central positive role in the acclimation to increased ambient temperature. Previously, *PIF4* was demonstrated to control the morphological acclimation to warm temperatures via auxin. *PIF4* bound to the promoters of the key auxin biosynthesis genes in a temperature-dependent manner [152,153]. Moreover, *PIF4* directly or indirectly stimulated the expression of auxin target *SMALL AUXIN UP RNA* (*SAUR*) 19–24 genes, which drive warmth-induced hypocotyl elongation and likely petiole elongation and leaf hyponasty [152]. Finally, many miRNAs and candidate genes have been identified to reduce the harmful effects of temperature stresses.

### 7.4. Heavy Metal Stress

At the present time, the world contains fast growing technology and industrialization, and the toxicity of heavy metals (Iron, Fe; arsenate, As; cadmium, Cd; chromium, Cr; lead, Pb; copper, Cu; mercury, Hg; and aluminum, Al) has developed as a global threat for all human beings. The accumulation of heavy metals causes devastation to the fertility of agricultural lands. Additionally, heavy metals are important to life only when they are present in a trace amount [154]. The presence of heavy metals in an excess amount is toxic to plant cells [155]. The excess amount of these heavy metals not only disturbs the plant kingdom, but also affects the animal kingdom. Their damaging impact on our agriculture has also been very well-documented [156,157].

**Figure 9:**
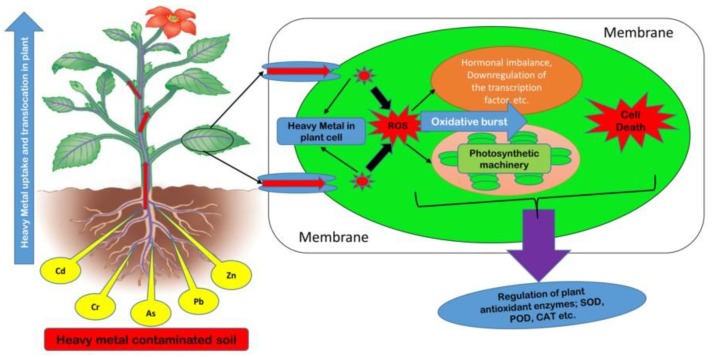
The mechanism of metal stress and oxidative damage in plant cells. Plants uptake an excess amount of heavy metals, which leads to the overproduction of ROS. These ROS cause an oxidative burst and affect a variety of plant biochemical activities in the plant cell.

At a cellular level, the elevated quantity of heavy metals imposes damage by a wide number of mechanisms. The most common mechanism is the production of reactive oxygen species (ROS) inducing oxidative stress, while others include the inactivation of biomolecules by the displacement of essential metal ions or by blocking essential functional groups [8]. Previous studies suggested that heavy metals acted as exchangers of essential metal ions or by blocking functional groups and caused oxidative damage at the cellular level. Metals like Fe and Cu, which are redox active, generated ROS directly through redox reactions; in contrast, other metals like Pb, Cd, Ni, Al, Mn, and Zn generated ROS through indirect mechanisms (Figure 9) [2].

Previous studies suggested that the toxicity of heavy metals disturbs redox homeostasis due to the overproduction of ROS such as singlet oxygen (^1^O_2_), superoxide radicals (O_2_^−^), hydrogen peroxide (H_2_O_2_), and hydroxyl radicals (^•^OH) [8]. The plant’s molecular response to heavy metal stress is characterized by the synthesis of stress-related amino acids, protein, genes, and signaling molecules [140]. A higher proline (Pro) level was found in the Cd-hyperaccumulator plant *Solanum nigrum* more than in the non-accumulator plant (*Solanum melongena* L), indicating its role in heavy metal detoxification [158,159].

The different kinetics of mitogen-activated protein kinase (MAPK) cascades in response to metal stress have also been reported [160]. The seedlings of *Medicago sativa* being exposed to Cd/Cu stress resulted in the activation of MAPK genes, including *SAMK*, *MMK2*, *MMK3*, and *SIMK*. Importantly, Cu stress rapidly activated *SIMK*, *MMK2*, *MMK3*, and *SAMK*, while Cd showed a similar but delayed MAPK activation [161,162].

Similarly, calcium signaling, hormone signaling, and MAPK signaling networks also demonstrated a positive response to the toxic effect of heavy metal stresses [161]. Calcium signaling employs a multitude of calcium sensing proteins like calmodulins (CaMs), CaM-like proteins (CMLs), calcineurin B-like proteins (CBLs), and Ca^2+^-dependent protein kinases (CDPKs) that bind to Ca^2+^ and trigger different downstream signaling pathways [162].

Another mechanism, cation diffusion facilitators (CDFs), also known as the metal tolerance protein (MTP) family, is reported in a diverse group of organisms such as bacteria, fungi, animals, and plants [163]. Twelve and ten MTP genes have been recognized so far in Arabidopsis and rice, respectively [164]. In Arabidopsis, the first CDF gene was characterized as the *Zinc Transporter 1* gene (*ZAT1*) and later renamed as *METAL TOLERANCE PROTEIN 1* (*AtMTP1*) [165]. The *AtMTP1* gene is expressed constitutively in the roots as well as in the shoots, and when overexpressed in Arabidopsis, it enhances Zn tolerance (Supplementary Table S4) [166,167,168].

In *A. halleri*, a Zn hyperaccumulator plant, the *AhMTP1* gene is believed to have a role in Zn hyper-tolerance [167]. Unlike the *AtMTP1* gene, *AtMTP3* is expressed predominantly in the roots and is reported to be engaged in the maintenance of Zn homeostasis by excluding Zn under Zn oversupply [168,169,170]. Another member of the MTP family, *AtMTP11*, has been reported to transport and to provide Mn tolerance [171]. In rice, an ortholog of MTPs, *OsMTP1*, was characterized and is thought to be located on chromosome 5; it is highly expressed in the mature leaves and stem [172,173].

Recently, it was reported that, in response to metal stress, plants regulate the location and accumulation of auxin by the differential and dynamic expression of auxin-related genes, like *phosphoribosyl anthranilate transferase 1* (*PAT1*), *CYP79B2* and *CYP79B3*, *YUCCA* (*YUC*), Gretchen Hagen (GH3) genes (TIR1), the PIN family, and the ABCB family [156]. Cu^2+^ toxicity in Arabidopsis led to changes in auxin and cytokinin accumulations and mitotic activity within the primary and secondary root tips [174]. Moreover, *aux1-7* and *pin2* mutants showed a high tolerance to Fe stress as compared to wild-type plants, which suggested that AUX and PIN proteins are potentially involved in the protection of lateral root formation under Fe stress [175].

Apart from this, Cd disrupts the maintenance of auxin homeostasis in Arabidopsis seedlings by increasing the IAA (indole-3-acetic acid) oxidase activity and altering the expression of several auxin biosynthetic and catabolic genes [176,177]. The Cd-mediated up-regulation of biosynthesis gene *NITRILASE* (*NIT*) resulted in increasing the IAA concentration in Arabidopsis roots promoting lateral root growth, thus protecting the roots from Cd [178]. Moreover, a recent report revealed the inhibition of root meristem growth through Cd-induced NO accumulation, which in turn repressed auxin transport and stabilized AUX/IAA proteins to repress auxin signaling [179]. A positive role for auxin transport through AUX1 on plant tolerance to As stress via ROS-mediated signaling was also disclosed in a study [180].

## 8. Recent Progresses in Genome Editing for Crop Improvement

Genome editing has become a very useful tool for crop improvement, and the advancements of CRISPR/Cas9 have significantly sped up crop breeding [11,12]. Recently, a great improvement in plants genome editing has been reported. Here, we will discuss some important progress for genome editing. Single gene traits are considered to be phenotypes controlled by a single gene. During the mutation process, these genes typically affect a specific trait without compromising other agronomic characteristics, making genome-editing tools especially suitable. For example, *AtNAC2* demonstrated an improved tolerance under limited water conditions without affecting the yield and other traits [95,102].

On the other hand, conventional breeding is unable to control/work on a single gene and is also time consuming. It is difficult to reduce the accumulation of cadmium in rice grains using traditional breeding methods, which can have serious health consequences for consumers. CRISPR/Cas9 has recently been used to knock out the metal transporter gene *OsNramp5* [52], which significantly reduced the concentration of Cd in seeds without significantly affecting yield. In Arabidopsis, *AhMATP1* and *AtMATP1* overexpression lines significantly enhanced the Zn tolerance [166,167]. The Fe stress tolerance in Arabidopsis was enhanced in *aux1-7* and *pin2* mutants [173,174,175,176,177,178].

Similarly, the other traits, such as flowering, height, and yield, were able to be controlled by a single gene. The FT2a in soybean can delay flowering time under both short-day and long-day conditions [179], thereby adapting the transgenic mutant plants to a larger geographic extent of the growing region [52]. Some traits, such as yield, are controlled by a complex network of genes or family. To overcome abiotic stress tolerance, a number of micro RNAs (miR319, miR169, miR398, miR169c, miR408, miR398a/b, etc.) have been identified as involved in a wide range of abiotic (cold, drought, and salinity) stress tolerances using CRISPR-Cas9 [47,49,50].

Drought is considered a key hurdle for crop production. Genome editing technology and plant biotechnology played a leading role in identifying candidate genes not only to enhance stress tolerance but also to improve crop production. *SnRK2* phosphorylates *KATs* and *SLAC1* to facilitate the stomatal opening to induce drought stress tolerance [90,91,92]. Similarly, the NAC transcription factor is involved in plant growth, development, and defense mechanisms. Recently, an OsNAC6 overexpression line was reported to regulate the root architecture and increase drought stress tolerance [95], while *OsNAC10* and *OsNAC5* not only induced drought stress tolerance but also facilitated the grain yield in rice [96,99,100,101].

High affinity potassium transporter (HKT) genes, such as *OsHKT1;1*, *OsHKT1;4*, and *OsHKT1;5*, facilitated salinity stress tolerance in rice [127,128]. Additionally, *TaHKT2;1*, *AtSOS1*, *OsCIPK24*, and *OsCBL4* were reported in different plant species in the regulation of salinity stress tolerance. Likewise, several transcription factors and micro-RNAs have been reported related to temperature stress tolerance. In rice, miR319, miR535, miR396b, and *ONAC095* were reported to increase cold stress tolerance [47,48,49,147]. Additionally, PIF4 is a key facilitator for auxin biosynthesis and is also required for hypocotyl elongation and high temperature stress tolerance [131].

Xing and Zhang (2010) demonstrated that rice grain yield can be controlled by many quantitative trait loci (QTL) [178] and reported that independent or multiplex editing of these QTL can result in an improved yield [76]. The editing of the same yield-related QTL in different elite rice varieties can have inconsistent or even negative effects under field conditions [27,179]. Thus, the ability to incorporate some complex traits, which cannot be controlled using conventional breeding techniques, can be controlled by genome editing technology.

Recently, two innovative rapid-breeding approaches, IMGE (haploid-inducer mediated genome editing) and Hi-Edit (haploid induction-edit), which combine haploid induction with CRISPR/Cas9-mediated genome editing was used to introduce desirable traits into elite inbred lines within two generations, avoiding the time-consuming crossing and back-crossing processes [180]. The MiMe phenotype in rice can be reproduced by the simultaneous editing of *OsSPO11-1*, *OsREC*8, and *OsOSD1*, suggesting that different sets of genes involved in meiosis can be manipulated to create the same phenotype [181,182]. Thus, an important benefit of genome editing tools is the ability to integrate complex features that cannot be introduced through traditional enhancement techniques. Moreover, plant biotechnology plays an important role in the genome editing of crops toward improvement for the benefit of humanity.

## 9. Conclusions

In the last two decades, numerous achievements have been reported in different plant species (rice, maize, tomato, tobacco, Arabidopsis, etc.) using CRISPR/Cas9 for genome editing. To have a successful impact on agricultural production under environmental stresses, more efforts are required to enhance and improve the CRIPSR/Cas9 technology to produce easy, approachable, and accessible methods for researchers.

Plant abiotic stresses are a major threat to crop production around the world, and these effects are also predicted to increase in the future. As mentioned above, plant responses to abiotic stresses are through very complex signaling pathways, and determining the genes involved and untangling the responses for practical application requires a multi-pronged approach. During the last two decades, plant abiotic stress has become a key issue for researchers to identify candidate genes and transcriptional factors. Several transcription factors (NAC, ARF, MYB, SOC, MAPK, CBFs, etc.) have been identified to overcome abiotic stresses, including low and high temperatures, drought stress, and heavy metal stress.

Transgenic breeding can create new and significant sources of resistance with rapid multiplication potentials, for instance, CRISPR/Cas9. The genome-edited crops through CRISPR/Cas9 should be considered as BE (bioengineered) crops for the quick application and acceptance of the technology in the field. We predict that CRISPR/Cas9 technology application in numerous plant species could revolutionize agriculture in a second green revolution, which could ensure the food demands and nutritional security are met for the increasing populations. As mentioned above, the global population is increasing rapidly, and this leads to enhanced food consumption. By using genome editing technologies, we can offer improved crop production to meet the demand for food. We should grasp this opportunity to increase crop production and the potential to save the lives of millions of people who are facing food shortages around the globe, especially in developing nations.

## Figures and Tables

**Table 1:** List of miRNAs involved in plant growth and developments.

miRNA	Species	Targeted Genes	Function	References
miR172a	Soybean	*SSAC1*	Salt tolerance	[52]
miR528	Maize	*ZmLAC3*, *ZmLAC5*	Lignin biosynthesis	[53]
miR319	Arabidopsis	*TCP4*	Leaf development and hormone biosynthesis and signaling	[54]
miR171c	Arabidopsis	*SCL6-II*, *SCL6-III*, *SCL6-IV*	Shoot and branching	[55]
miR319	Rice	*TCP21*, JA biosynthesis and signaling-related genes (*PLDα1*, *LOX5*, *LOX11*, *CORI1b* and *CORI2*)	Rice ragged stunt virus resistance	[56]
miR528	Rice	*OsSPL9*	Rice stripe virus resistance	[57]
miR528	Rice	*OsRFI2*	Flowering time	[58]
miR156	Brassica	*BrpSPL9-2*	Control heading time	[59]
miR157b		*SPL*	Control of various developmental process	[60]
miR159	Arabidopsis	*AtMY*B	Involved in GA signaling pathway and control programmed cell death and flowering	[61]
miR393	Rice	*OsAFB2, OsTIR1*	Early flowering, salt and drought stress tolerance	[62]
miR169	Arabidopsis	*NY-YA2*	Promote flowering and stress tolerance	[63]
miR171	Arabidopsis	*SPL*	Control plant growth and flowering time	[64]
miR159	Rice	*OsGAMYBLs*	Regulate heading	[65]
miR172	Maize	*ZmTOE1*	Regulate flowering time	[66]
miR169	Arabidopsis	*PH2*	Abiotic stress response	[67]
miR159	Tobacco	*GAMYB*	Pathogen defense response	[68]

## Supplementary Materials

Supplementary materials can be found at https://www.mdpi.com/1422-0067/21/8/2695/s1.
