# Supplementary material for: Transgenic Breeding Approaches for Improving Abiotic Stress Tolerance: Recent Progress and Future Perspectives
Source: Int J Mol Sci. 2020 Apr 13;21(8):2695. doi: 10.3390/ijms21082695 (PMC7216248; doi:10.3390/ijms21082695)
Supplement: Supplementary file 1 [file ijms-21-02695-s001.pdf]

Review

Transgenic Breeding Approaches for Improving Abiotic Stress Tolerance: Recent Progress and Future Perspectives

Ali Anwar and Ju-Kon Kim*

Graduate School of International Agricultural Technology and Crop Biotechnology Institute/GreenBio Science & Technology, Seoul National University, Pyeongchang 25354, Korea; anwar_ali@snu.ac.kr

* Correspondence: jukon@snu.ac.kr

Table 1. List of drought response genes in plants.

Genes	Species	Remarks	Reference
TaDREB3	Wheat	Dehydration-responsive element-binding 3 increase yield under limited water condition.	[1]
Cu/Zn-SOD	Tobacco/Rice	Cu/Zn-superoxide dismutase, involved in drought stress tolerance, through activation plant defense system to control overproduction of ROS.	[2]
MuWRKY3	Groundnut	*MuWRKY3* regulate antioxidant (SOD, CAT, APX) enzyme activates and showed less accumulation of MDA and ROS in groundnut under drought stress.	[3]
TPS1	Potato	*Trehalose-6-phosphate synthase 1*, a potato gene regulate drought stress tolerance, as well as Sugars, starch and proline accumulation.	[4]
HSI2	Arabidopsis	*SUGAR INDUCIBLE GENE 2*, is also known as *VAL1* that reduced wilting and maintained higher relative water contents, and displayed lower constitutive and ABA-induced stomatal conductance to regulate drought stress tolerance	[5]
SUMO	Arabidopsis	A Small Ubiquitin related Modifier (*SaSCe9*), regulate the transcript level of *AtSOD*, *AtCAT*, *AtNHX1*, *AtSOS1* to regulation under drought stress	[6]
OsCPK9	Rice	A calcium-dependent protein kinases gene, played positive role in enhancing stomatal closure and improving the osmotic adjustment ability.	[7]
miR394 & LCR	Arabidopsis	*LCR* and *miR394* are essential for maintain or enhance Salt and drought stress	[8]
LcMYB2	Sheepgrass	A MYB-related transcription factor, regulate the expression *AtDREB2A*, *AtLEA14* and *AtP5CS1* genes to increase resistance to drought stress.	[9]
ONAC095	Rice	NAC transcriptional factors, involved widely in abiotic stresses. The *ONAC095-SRDX* plants displayed an improved drought tolerance, decreased water loss rate, increased proline and soluble sugar contents, and up-regulated expression of drought-responsive genes under drought stress.	[10]
OsGRAS23	Rice	Located on Chr 4 in rice that's induce drought stress tolerance. *OsGRAS23* encodes a stress-responsive GRAS transcription factor and positively modulates rice drought tolerance via the induction of a number of stress-responsive genes	[11]
MaPIP1;1	Banana	*MaPIP1;1* in Arabidopsis confers salt and drought stress tolerances by reducing membrane injury, improving ion distribution and maintaining osmotic balance.	[12]
OsbZIP62	Rice	*OsbZIP62* is involved in ABA signaling pathways, and positively regulates rice drought tolerance	[13]
OsERF71	Rice	*OsERF71*, predominantly express in root meristem, pericycle and endodermis, conferring drought resistance at the reproductive stage and also regulate grain yield.	[14]
OsNAC5	Rice	*OsNAC5* overexpressing lines in rice induce drought stress tolerance in rice and also regulate grain yield	[15]

OsNAC 9	Rice	*OsNAC9* alters the root architecture and yield, and involved in drought stress tolerance.	[16]

Table S2. List of salinity stress response genes in plants

Genes	Species	Remarks	Reference
AtSKIP	Arabidopsis	*SKIP* is involved in self-transcriptional activation activity, works as positive regulator and putative potential transcription factor abiotic stress tolerance.	[17]
OsHSP71.0 & *23.7*	Rice	*HSPs* is broad family is known as heat shock protein, regulate drought and salinity stress tolerance in rice.	[18]
OsNHXs	Rice	*OsNHX1* localized on tonoplast, play an important role in Na^+ and K^+ regulation and enhanced salinity tolerance and growth in rice.	[19]
TaNHX2	Wheat	Regulate salinity stress tolerance and growth performance, chlorophyll content, increased proline accumulation and control ROS production through activation defense system.	[20]
SAPK1 and *SAPK2*	Rice	Involved in salinity tolerance, seed germination, chlorophyll accumulation, activate antioxidant enzymes activities and reduce overproduction of ROS.	[21]
HvHKT2;1	Barley	increase plant response to salt stress tolerance and growth performance.	[22]
miR172c	Soybean	Overexpression *miR172* resulted a significant increase in root activity, growth and tolerance to salt stress.	[23]
OsJAZ8	Rice	*OsJAZ8* is negative regulator of Jasmonate signaling pathway, that's are involved in salt stress tolerance and also control number developmental characteristics in japonica rice.	[24]
GsMAPK4	Soybean	The overexpression *GsMAPK4* induce salinity stress tolerance through activating defense system.	[25]
OsSNAC1	Rice	Enhancing root development and reducing transpiration rate, biochemical adjustment	[26]
OsRab7	Rice	Vesicle trafficking gene enhanced seedling growth and increased proline content	[27]
PtSOS2	Poplar	Protein kinases enhanced photosynthetic pigments and physiological parameters	[28]
PeXTH	Poplar	Regulate number of developmental process, cell viability and membrane stability enhanced water holding capacity.	[29]
ONAC045	Rice	Functioned as a transcriptional activator.	[30]
TaSTRG	Rice	Higher salt and drought tolerance, lower intracellular Na^+/K^+ ratio, higher survival rate, fresh weight and chlorophyll content, accumulated higher proline and soluble sugar contents and had significantly higher expression levels of putative proline synthetase and transporter genes	[31]
AtSTO1	Poplar	Increased concentrations of 9-cis-epoxycarotenoid dioxygenase3, greater overall biomass, greater root biomass, improved photosynthesis.	[32]

DcHsp17.7	Carrot	Involved in stress regulatory mechanism, cell viability and membrane stability under heat stress.	[33]
OsHKT1;5	Rice	*OsHKT1;5* regulate rice growth and development, and induce salinity stress tolerance and protect overproduction of ROS and oxidative damage.	[34]
TmHKT1; 5-A, 1;4-A2	Wheat	*TmHKT1;5-A* and *TaHKT1;5-D* encode dual affinity Na^+ transporters, can be blocked by raising the external K^+ concentration, leads to increase salinity stress tolerance.	[35]
SbHKT1;4, HvHKT1 & 2	Sorghum & barley	Involved in enhancing salinity stress tolerance and activate plant defense mechanism.	[36, 37]
SOS1, 2, 3	Sorghum	Mediate cellular signaling under salt stress, to maintain ion homeostasis, regulatory mechanisms and response to salt stress. *SOS3* also plays a critical role in plastic development of lateral roots through modulation of auxin gradients and maxima in roots under mild salt conditions.	[38]
SOS2	Arabidopsis	*Salt Overly Sensitive 2* (*SOS2*) gene, involved in Na^+/K^+ hemostasis under salinity stress	[39]
OsSOS1	Rice	Salt Overly Sensitive gene, regulate salt stress tolerance	[40]
ZmWRKY17	Arabidopsis	*WRKY* is widely involved in abiotic stress, through ABA signal activation and regulation, and MDA accumulation to protect ROS overproduction	[41]

Table S3. List of temperature stress response genes in plants

Genes	Species	Remarks	Reference
CBF	Arabidopsis	*CBF* involved in cold stress tolerance. Protect plant cell from oxidative stress, through activating defense system in plants.	[42]
CBF/DREB1	Arabidopsis	*CBF/DREB1* is TF, involved in freezing stress tolerance, and regulate JASMONATE biosynthesis and signaling pathway.	[43]
CBF14	Wheat & Barley	A key components of the light quality-dependent regulation of the freezing tolerance, through integration of phytochrome-mediated light and temperature signaling pathway.	[44]
HY5	Arabidopsis	*HY5* positively regulates cold induced gene expression through Z-box/LTRE and hy5 mutant is sensitive	[45]
PhyB	Arabidopsis	*Phytochrome B* mutant induce cold tolerance, decrease electrolyte leakage, MDA, upregulate *DREBs* gene expression.	[46]
PRR5/7/9	Arabidopsis	acts as a negative regulators for *CBF* pathway and thus, triple mutant *prr5/7/9* showed increased cold tolerance	[47]

CCA1	Arabidopsis	*CCA1* binds to promoter of *CBFs* and promotes cold acclimation which is self-regulated in response to cold stress by alternative splicing mechanism	[48]
TaDREB3	Barley	TaDREB3, is cold inducible promoter in plants. Its regulate cold stress tolerance, and improve plant worth and yield	[49]
OsDREBs	Rice	*OsDREB* induce clod stress tolerance, Over-expression of *OsDREB1A* in transgenic Arabidopsis induced over-expression of target stress-inducible genes of Arabidopsis *DREB1A* resulting in plants with higher tolerance to drought, high-salt, and freezing stresses.	[50]
TaPEPKR2	Wheat	*TaPEPKR2*, is phosphoenolpyruvate carboxylase kinase-related kinase gene involved in Heat stress tolerance and also regulate defense related gene in wheat.	[51]
TaFBA1	Wheat	*TaFBA1*, encodes a homologous F-box protein, can induce heat stress (45˚C) tolerance. It's also stabilize ROS accumulation, Antioxidant enzymes activities, chlorophylls and sugar accumulation.	[52]
SNAC3	Rice	*SNAC3* may confer stress tolerance (Heat, Cold, ABA) by modulating reactive oxygen species (ROS) homeostasis, as well as regulate plant growth and developments.	[53]
sHSP26	Arabidopsis	*HSP23.9* is small heat shock proteins induce heat and salt stress tolerance.	[54]
CsCaM3	Cucumber	CsCaM3 induce heat stress tolerance and protect oxidative damage, improve chlorophyll, photosynthetic capacity and heat stress response gene	[55]
SbDhn1	Sorghum bicolor	*SbDHN1* lines significantly increase chlorophylls, soluble sugar, and tolerance to heat stress	[56]
csa-miR159b	Cucumber	*csa-miR159b* involved in abscisic acid (ABA) biosynthesis, and interact with MY and HSP under heat stress tolerance	[57]
CsWRKY46	Cucumber	*CsWRKY46*, is belong to *WRKY* gene family, potentially involved in Cold stress tolerance	[58]

Table S4. List of Heavy metal stress response genes in plants

Genes	Species	Remarks	Reference
AtFC1	Arabidopsis	*Ferrochelatase-1* (*AtFC1*), involved in Cd stress tolerance. *AtFC1* overexpression lines increase root elongation, chlorophylls, and antioxidant enzymes activates under Cd stress.	[59]
JrVHAG1	Walnut	*JrVHAG1* gene functions as a Cd stress response regulator by participating in ABA-signal pathway and *MYB* transcription regulation network.	[60]
OsZIP1	Rice	*OsZIP1*, a metal efflux transporter that is required for detoxification of excess Zn, Cu and Cd in rice.	[61]
OsMT1a	Rice	*OsMT1a*, is a type Metallothionein involved in zinc hemostasis, and regulate heavy metal stress tolerance in rice.	[62]
OsATX1	Rice	Antioxidant Protein1 (*OsATX1*), overexpression reduced Cu accumulation in roots, and shows a strong resistance to Cu stress through activation stress response genes.	[63]
Nramp5	Rice	As major Mg and Cd transporter and also induce stress tolerance	[64]
OsHMA5	Rice	*OsHMA5* is involved in copper xylem loading in Rice	[65]
OsYSL2	Rice	*OsYSL2*, is required for the long-distance transport of Fe and Mg.	[66]
ZmYS1	Maize	Maize yellow stripe 1 (*ZmYS1*), encoding membrane protein, involved in iron uptake from soil and leading to chlorosis, improve yield and quality.	[67]
HY1	Arabidopsis	Arabidopsis *HY1* involved in iron hemostasis, decrease nitric oxide biosynthesis and increase Cd stress tolerance.	[68]
AtMTP11	Arabidopsis	*MTP* family of genes encode proteins of the cation diffusion facilitator (CDF) family, and involved in Mn stress tolerance.	[69]
AhMTP1	Arabidopsis	Metal Tolerance Protein 1 , Zn_2^+/H^+ antiporter involved in cytoplasmic zinc detoxification to induce Zn stress tolerance	[70]
OsMTP1	Rice	*OsMTP1*, cation transporter localized in the cell membrane, involved in tolerance to Zn, Cd, and Ni, and also improve rice growth.	[71]

Reference

1. Shavrukov, Y.; Baho, M.; Lopato, S.; Langridge, P., The *TaDREB3* transgene transferred by conventional crossings to different genetic backgrounds of bread wheat improves drought tolerance. *Plant Biotechnol. J.* **2016**, *14*, 313–322.
2. Hamid Badawi, G.; Yamauchi, Y.; Shimada, E.; Sasaki, R.; Kawano, N.; Tanaka, K.; Tanaka, K., Enhanced tolerance to salt stress and water deficit by overexpressing superoxide dismutase in tobacco (*Nicotiana tabacum*) chloroplasts. *Plant Sci.* **2004**, *166*, 919–928.
3. Kiranmai, K.; Lokanadha Rao, G.; Pandurangaiah, M.; Nareshkumar, A.; Amaranatha Reddy, V.; Lokesh, U.; Venkatesh, B.; Anthony Johnson, A.M.; Sudhakar, C., A Novel WRKY Transcription Factor, MuWRKY3 (*Macrotyloma uniflorum* Lam. Verdc.) enhances drought stress tolerance in transgenic groundnut (*Arachis hypogaea* L.) Plants. *Front. Plant Sci.* **2018**, *9*, 346.
4. Kondrák, M.; Marincs, F.; Antal, F.; Juhász, Z.; Bánfalvi, Z., Effects of yeast trehalose-6-phosphate synthase 1 on gene expression and carbohydrate contents of potato leaves under drought stress conditions. *BMC Plant Biol.* **2012**, *12*, 74.
5. Sharma, N.; Bender, Y.; Boyle, K.; Fobert, P.R., High-level expression of *sugar inducible gene2* (*HSI2*) is a negative regulator of drought stress tolerance in Arabidopsis. *BMC Plant Biol.* **2013**, *13*, 170.
6. Karan, R.; Subudhi, P.K., A stress inducible SUMO conjugating enzyme gene (*SaSce9*) from a grass halophyte Spartina alterniflora enhances salinity and drought stress tolerance in Arabidopsis. *BMC Plant Biol.* **2012**, *12*, 187.
7. Wei, S.; Hu, W.; Deng, X.; Zhang, Y.; Liu, X.; Zhao, X.; Luo, Q.; Jin, Z.; Li, Y.; Zhou, S.; et al. A rice calcium-dependent protein kinase *OsCPK*9 positively regulates drought stress tolerance and spikelet fertility. *BMC Plant Biol.* **2014**, *14*, 133.
8. Song, J.B.; Gao, S.; Sun, D.; Li, H.; Shu, X.X.; Yang, Z.M., *miR394* and *LCR* are involved in Arabidopsis salt and drought stress responses in an abscisic acid-dependent manner. *BMC Plant Biol.* **2013**, *13*, 210.
9. Zhao, P.; Hou, S.; Guo, X.; Jia, J.; Yang, W.; Liu, Z.; Chen, S.; Li, X.; Qi, D.; Liu, G.; et al. A MYB-related transcription factor from sheepgrass, *LcMYB2*, promotes seed germination and root growth under drought stress. *BMC Plant Biol.* **2019**, *19*, 564.
10. Huang, L.; Hong, Y.; Zhang, H.; Li, D.; Song, F., Rice NAC transcription factor *ONAC095* plays opposite roles in drought and cold stress tolerance. *BMC Plant Biol.* **2016**, *16*, 203.
11. Xu, K.; Chen, S.; Li, T.; Ma, X.; Liang, X.; Ding, X.; Liu, H.; Luo, L., *OsGRAS*23, a rice GRAS transcription factor gene, is involved in drought stress response through regulating expression of stress-responsive genes. *BMC Plant Biol.* **2015**, *15*, 141.
12. Xu, Y.; Hu, W.; Liu, J.; Zhang, J.; Jia, C.; Miao, H.; Xu, B.; Jin, Z., A banana aquaporin gene, *MaPIP1;1*, is involved in tolerance to drought and salt stresses. *BMC Plant Biol.* **2014**, *14*, 59.
13. Yang, S.; Xu, K.; Chen, S.; Li, T.; Xia, H.; Chen, L.; Liu, H.; Luo, L., A stress-responsive bZIP transcription factor *OsbZIP62* improves drought and oxidative tolerance in rice. *BMC Plant Biol.* **2019**, *19*, 260.
14. Lee, D.-K.; Jung, H.; Jang, G.; Jeong, J.S.; Kim, Y.S.; Ha, S.-H.; Choi, Y.D.; Kim, J.-K., Overexpression of the *OsERF71* transcription factor Alters Rice Root Structure and Drought Resistance. *Plant Physiol.* **2016**, *172*, 575–588.
15. Jeong, J.S.; Kim, Y.S.; Redillas, M.C.F.R.; Jang, G.; Jung, H.; Bang, S.W.; Choi, Y.D.; Ha, S.-H.; Reuzeau, C.; Kim, J.-K., OsNAC5 overexpression enlarges root diameter in rice plants leading to enhanced drought tolerance and increased grain yield in the field. *Plant Biotechnol. J.* **2013**, *11*, 101–114.
16. Redillas, M.C.F.R.; Jeong, J.S.; Kim, Y.S.; Jung, H.; Bang, S.W.; Choi, Y.D.; Ha, S.-H.; Reuzeau, C.; Kim, J.-K., The overexpression of *OsNAC9* alters the root architecture of rice plants enhancing drought resistance and grain yield under field conditions. *Plant. Biotechnol. J.* **2012**, *10*, 792–805.
17. Lim, G.-H.; Zhang, X.; Chung, M.-S.; Lee, D.J.; Woo, Y.-M.; Cheong, H.-S.; Kim, C.S., A putative novel transcription factor, *AtSKIP*, is involved in abscisic acid signalling and confers salt and osmotic tolerance in Arabidopsis. *New Phytol.* **2010**, *185*, 103–113.

18. Zou, J.; Liu, C.; Liu, A.; Zou, D.; Chen, X., Overexpression of *OsHsp17.0* and *OsHsp23.7* enhances drought and salt tolerance in rice. *J. Plant. Physiol.* **2012**, *169*, 628–635.
19. Fukuda, A.; Nakamura, A.; Tagiri, A.; Tanaka, H.; Miyao, A.; Hirochika, H.; Tanaka, Y., Function, intracellular localization and the importance in salt tolerance of a vacuolar $Na(^+)/H(^+)$ antiporter from rice. *Plant. Cell Physiol.* **2004**, *45*, 146–159.
20. Mushke, R.; Yarra, R.; Kirti, P.B., Improved salinity tolerance and growth performance in transgenic sunflower plants via ectopic expression of a wheat antiporter gene (*TaNHX2*). *Mol. Bio. Rep.* **2019**, *46*, 5941–5953.
21. Lou, D.; Wang, H.; Yu, D., The *sucrose non-fermenting-1-related protein kinases SAPK1* and *SAPK2* function collaboratively as positive regulators of salt stress tolerance in rice. *BMC Plant. Biol.* **2018**, *18*, 203.
22. Mian, A.; Oomen, R.J.F.J.; Isayenkov, S.; Sentenac, H.; Maathuis, F.J.M.; Véry, A.-A., Over-expression of an $Na^+$and K^+permeable HKT transporter in barley improves salt tolerance. *Plant., J.* **2011**, *68*, 468–479.
23. Sahito, Z.A.; Wang, L.; Sun, Z.; Yan, Q.; Zhang, X.; Jiang, Q.; Ullah, I.; Tong, Y.; Li, X., The *miR172c-NNC1* module modulates root plastic development in response to salt in soybean. *BMC Plant. Biol.* **2017**, *17*, 229.
24. Peethambaran, P.K.; Glenz, R.; Höninger, S.; Shahinul Islam, S.M.; Hummel, S.; Harter, K.; Kolukisaoglu, Ü.; Meynard, D.; Guiderdoni, E.; Nick, P.; Riemann, M., Salt-inducible expression of *OsJAZ8* improves resilience against salt-stress. *BMC Plant. Biol.* **2018**, *18*, 311.
25. Qiu, Y.-w.; Feng, Z.; Fu, M.-m.; Yuan, X.-h.; Luo, C.-c.; Yu, Y.-b.; Feng, Y.-z.; Wei, Q.; Li, F.-l., *GsMAPK4*, a positive regulator of soybean tolerance to salinity stress. *J. Integr. Agri.* **2019**, *18*, 372–380.
26. Liu, G.; Li, X.; Jin, S.; Liu, X.; Zhu, L.; Nie, Y.; Zhang, X., Overexpression of rice NAC gene *SNAC1* improves drought and salt tolerance by enhancing root development and reducing transpiration rate in transgenic cotton. *PLoS ONE* **2014,** *9*, e86895.
27. Peng, X.; Ding, X.; Chang, T.; Wang, Z.; Liu, R.; Zeng, X.; Cai, Y.; Zhu, Y., Overexpression of a Vesicle Trafficking Gene, *OsRab7*, enhances salt tolerance in rice. *Sci. World, J.* **2014**, *2014*, 483526. https://doi.org/10.1155/2014/483526.
28. Zhou, J.; Wang, J.; Bi, Y.; Wang, L.; Tang, L.; Yu, X.; Ohtani, M.; Demura, T.; Zhuge, Q., Overexpression of *PtSOS2* enhances salt tolerance in transgenic poplars. *Plant. Mol. Bio. Rep.* **2014**, *32*, 185–197.
29. Han, Y.; Wang, W.; Sun, J.; Ding, M.; Zhao, R.; Deng, S.; Wang, F.; Hu, Y.; Wang, Y.; Lu, Y.; et al. Populus euphratica *XTH* overexpression enhances salinity tolerance by the development of leaf succulence in transgenic tobacco plants. *J. Exp.Bot.* **2013**, *64*, 4225–4238.
30. Zheng, X.; Chen, B.; Lu, G.; Han, B., Overexpression of a *NAC* transcription factor enhances rice drought and salt tolerance. *Biochem. Bioph. Res. Co.* **2009**, *379*, 985–989.
31. Zhou, W.; Li, Y.; Zhao, B.C.; Ge, R.C.; Shen, Y.Z.; Wang, G.; Huang, Z.J., Overexpression of *TaSTRG* gene improves salt and drought tolerance in rice. *J. Plant. Physiol.* **2009**, *166*, 1660–1671.
32. Lawson, S.S.; Michler, C.H., Overexpression of *AtSTO1* leads to improved salt tolerance in *Populus tremula* x P. alba. *Transgenic Res.* **2014**, *23*, 817–826.
33. Song, N.H.; Ahn, Y.J., *DcHsp17.7*, a small heat shock protein in carrot, is tissue-specifically expressed under salt stress and confers tolerance to salinity. *New Biotechnol.* **2011**, *28*, 698–704.
34. Kobayashi, N.I.; Yamaji, N.; Yamamoto, H.; Okubo, K.; Ueno, H.; Costa, A.; Tanoi, K.; Matsumura, H.; Fujii-Kashino, M.; Horiuchi, T.; et al. *OsHKT1;5* mediates $Na(^+)$ exclusion in the vasculature to protect leaf blades and reproductive tissues from salt toxicity in rice. *Plant. J.* **2017**, *91*, 657–670.
35. Xu, B.; Hrmova, M.; Gilliham, M., High affinity Na^+ transport by wheat *HKT1;5* is blocked by K^+. *BioRxiv* **2018**, 280453, doi:https://doi.org/10.1101/28045.
36. Han, Y.; Yin, S.; Huang, L.; Wu, X.; Zeng, J.; Liu, X.; Qiu, L.; Munns, R.; Chen, Z.H.; Zhang, G., A Sodium Transporter *HvHKT1;1* confers salt tolerance in barley via regulating tissue and cell ion homeostasis. *Plant. Cell Physiol.* **2018**, *59*, 1976–1989.

37. Wang, T.T.; Ren, Z.J.; Liu, Z.Q.; Feng, X.; Guo, R.Q.; Li, B.G.; Li, L.G.; Jing, H.C., *SbHKT1;4*, a member of the high-affinity potassium transporter gene family from Sorghum bicolor, functions to maintain optimal $Na^{(+)}$ /$K^{(+)}$ balance under $Na^{(+)}$ stress. *J. Integr. Plant. Biol.* **2014**, *56*, 315–332.
38. Ji, H.; Pardo, J.M.; Batelli, G.; Van Oosten, M.J.; Bressan, R.A.; Li, X., The *Salt Overly Sensitive* (*SOS*) pathway: Established and emerging roles. *Mol. Plant.* **2013**, *6*, 275–286.
39. Liu, J.; Ishitani, M.; Halfter, U.; Kim, C.S.; Zhu, J.K., The *Arabidopsis thaliana SOS2* gene encodes a protein kinase that is required for salt tolerance. *P. Natl. Acad. Sci. USA* **2000**, *97*, 3730–3734.
40. Martinez-Atienza, J.; Jiang, X.; Garciadeblas, B.; Mendoza, I.; Zhu, J.K.; Pardo, J.M.; Quintero, F.J., Conservation of the salt overly sensitive pathway in rice. *Plant. Physiol.* **2007**, *143*, 1001–1012.
41. Cai, R.; Dai, W.; Zhang, C.; Wang, Y.; Wu, M.; Zhao, Y.; Ma, Q.; Xiang, Y.; Cheng, B., The maize WRKY transcription factor *ZmWRKY17* negatively regulates salt stress tolerance in transgenic Arabidopsis plants. *Planta* **2017**, *246*, 1215–1231.
42. Cook, D.; Fowler, S.; Fiehn, O.; Thomashow, M.F., A prominent role for the CBF cold response pathway in configuring the low-temperature metabolome of Arabidopsis. *P. Natl. Acad. Sci. USA* **2004,** 101, (42), 15243-15248.
43. Hu, Y.; Jiang, L.; Wang, F.; Yu, D., Jasmonate Regulates the Inducer of CBF Expression–C-Repeat Binding Factor/DRE Binding Factor1 Cascade and freezing tolerance in Arabidopsis. *Plant. Cell* **2013**, *25*, 2907–2922.
44. Novak, A.; Boldizsar, A.; Gierczik, K.; Vagujfalvi, A.; Adam, E.; Kozma-Bognar, L.; Galiba, G., Light and temperature signalling at the level of *CBF14* gene expression in wheat and barley. *Plant. Mol. Bio. Rep.* **2017**, *35*, 399–408.
45. Catalá, R.; Medina, J.; Salinas, J., Integration of low temperature and light signaling during cold acclimation response in Arabidopsis. *P. Natl. Acad. Sci. USA* **2011**, *108*, 16475–16480.
46. He, Y.; Li, Y.; Cui, L.; Xie, L.; Zheng, C.; Zhou, G.; Zhou, J.; Xie, X., Phytochrome B negatively affects cold tolerance by regulating *OsDREB1* gene expression through Phytochrome Interacting Factor-Like Protein *OsPIL16* in Rice. *Fron. Plant. Sci.* **2016**, *7*, 1963–1963.
47. Nakamichi, N.; Kusano, M.; Fukushima, A.; Kita, M.; Ito, S.; Yamashino, T.; Saito, K.; Sakakibara, H.; Mizuno, T., Transcript profiling of an Arabidopsis PSEUDO RESPONSE REGULATOR arrhythmic triple mutant reveals a role for the circadian clock in cold stress response. *Plant. Cell Physiol.* **2009**, *50*, 447–462.
48. Seo, P.J.; Park, M.-J.; Lim, M.-H.; Kim, S.-G.; Lee, M.; Baldwin, I.T.; Park, C.-M., A self-regulatory circuit of *CIRCADIAN CLOCK-ASSOCIATED1* underlies the circadian clock regulation of temperature responses in Arabidopsis. *Plant. Cell* **2012**, *24*, 2427–2442.
49. Kovalchuk, N.; Jia, W.; Eini, O.; Morran, S.; Pyvovarenko, T.; Fletcher, S.; Bazanova, N.; Harris, J.; Beck-Oldach, K.; Shavrukov, Y.; et al. Optimization of *TaDREB3* gene expression in transgenic barley using cold-inducible promoters. *Plant. Biotechnol. J.* **2013**, *11*, 659–670.
50. Dubouzet, J.G.; Sakuma, Y.; Ito, Y.; Kasuga, M.; Dubouzet, E.G.; Miura, S.; Seki, M.; Shinozaki, K.; Yamaguchi-Shinozaki, K., *OsDREB* genes in rice (*Oryza sativa* L.) encode transcription activators that function in drought-, high-salt- and cold-responsive gene expression. *Plant. J.* **2003**, *33*, 751–763.
51. Zang, X.; Geng, X.; He, K.; Wang, F.; Tian, X.; Xin, M.; Yao, Y.; Hu, Z.; Ni, Z.; Sun, Q.; et al. Overexpression of the wheat (*Triticum aestivum* L.) *TaPEPKR2* gene enhances heat and dehydration tolerance in both Wheat and Arabidopsis. *Front. Plant. Sci.* **2018**, *9*, 1710.
52. Li, Q.; Wang, W.; Wang, W.; Zhang, G.; Liu, Y.; Wang, Y.; Wang, W., Wheat F-Box protein gene *TaFBA1* is involved in plant tolerance to heat stress. *Front. Plant. Sci.* **2018**, *9*, 521.
53. Fang, Y.; Liao, K.; Du, H.; Xu, Y.; Song, H.; Li, X.; Xiong, L., A stress-responsive *NAC* transcription factor *SNAC3* confers heat and drought tolerance through modulation of reactive oxygen species in rice. *J. Exp. Bot.* **2015**, *66*, 6803–6817.
54. Khurana, N.; Chauhan, H.; Khurana, P., Wheat chloroplast targeted *sHSP26* promoter confers heat and abiotic stress inducible expression in transgenic Arabidopsis plants. *PLoS ONE* **2013,** *8*, e54418.

55. Yu, B.; Yan, S.; Zhou, H.; Dong, R.; Lei, J.; Chen, C.; Cao, B., Overexpression of *CsCaM3* improves high temperature tolerance in cucumber. *Front. Plant. Sci.***2018**, *9*, 797-797.
56. Halder, T.; Upadhyaya, G.; Ray, S., YSK2 Type Dehydrin (*SbDhn1*) from sorghum bicolor showed improved protection under high temperature and osmotic stress condition. *Front. Plant. Sci.* **2017**, *8*, 918.
57. Li, H.; Wang, Y.; Wang, Z.; Guo, X.; Wang, F.; Xia, X.J.; Zhou, J.; Shi, K.; Yu, J.Q.; Zhou, Y.H., Microarray and genetic analysis reveals that *csa-miR159b* plays a critical role in abscisic acid-mediated heat tolerance in grafted cucumber plants. *Plant. Cell Environ.* **2016**, *39*, 1790–1804.
58. Zhang, Y.; Yu, H.; Yang, X.; Li, Q.; Ling, J.; Wang, H.; Gu, X.; Huang, S.; Jiang, W., *CsWRKY46*, a *WRKY* transcription factor from cucumber, confers cold resistance in transgenic-plant by regulating a set of cold-stress responsive genes in an ABA-dependent manner. *Plant. Physiol. Biochem.* **2016**, *108*, 478–487.
59. Song, J.; Feng, S.J.; Chen, J.; Zhao, W.T.; Yang, Z.M., A cadmium stress-responsive gene *AtFC1* confers plant tolerance to cadmium toxicity. *BMC Plant. Biology* **2017**, *17*, 187.
60. Xu, Z.; Ge, Y.; Zhang, W.; Zhao, Y.; Yang, G., The walnut *JrVHAG1* gene is involved in cadmium stress response through ABA-signal pathway and MYB transcription regulation. *BMC Plant. Biol.* **2018**, *18*, 19.
61. Liu, X.S.; Feng, S.J.; Zhang, B.Q.; Wang, M.Q.; Cao, H.W.; Rono, J.K.; Chen, X.; Yang, Z.M., OsZIP1 functions as a metal efflux transporter limiting excess zinc, copper and cadmium accumulation in rice. *BMC Plant. Biol.* **2019**, *19*, 283.
62. Yang, Z.; Wu, Y.; Li, Y.; Ling, H.-Q.; Chu, C., OsMT1a, a type 1 metallothionein, plays the pivotal role in zinc homeostasis and drought tolerance in rice. *Plant. Mol. Bio.* **2009**, *70*, 219–229.
63. Zhang, Y.; Chen, K.; Zhao, F.-J.; Sun, C.; Jin, C.; Shi, Y.; Sun, Y.; Li, Y.; Yang, M.; Jing, X.; et al. *OsATX1* interacts with heavy metal P1B-Type ATPases and affects copper transport and distribution. *Plant. Physiol.* **2018**, *178*, 329–344.
64. Sasaki, A.; Yamaji, N.; Yokosho, K.; Ma, J.F., *Nramp5* is a major transporter responsible for manganese and cadmium uptake in rice. *Plant. Cell* **2012**, *24*, 2155–2167.
65. Deng, F.; Yamaji, N.; Xia, J.; Ma, J.F., A member of the heavy metal P-Type ATPase *OsHMA5* is involved in xylem loading of copper in rice. *Plant. Physiol.* **2013**, *163*, 1353–1362.
66. Ishimaru, Y.; Masuda, H.; Bashir, K.; Inoue, H.; Tsukamoto, T.; Takahashi, M.; Nakanishi, H.; Aoki, N.; Hirose, T.; Ohsugi, R.; et al. Rice metal-nicotianamine transporter, *OsYSL2*, is required for the long-distance transport of iron and manganese. *Plant. J.* **2010**, *62*, 379–390.
67. Curie, C.; Panaviene, Z.; Loulergue, C.; Dellaporta, S.L.; Briat, J.-F.; Walker, E.L., Maize yellow stripe1 encodes a membrane protein directly involved in Fe(III) uptake. *Nature* **2001**, *409*, 346–349.
68. Han, B.; Yang, Z.; Xie, Y.; Nie, L.; Cui, J.; Shen, W., Arabidopsis *HY1* confers cadmium tolerance by decreasing nitric oxide production and improving iron homeostasis. *Mol. Plant.* **2014**, *7*, 388–403.
69. Delhaize, E.; Gruber, B.D.; Pittman, J.K.; White, R.G.; Leung, H.; Miao, Y.; Jiang, L.; Ryan, P.R.; Richardson, A.E., A role for the AtMTP11 gene of Arabidopsis in manganese transport and tolerance. *Plant. J.* **2007**, *51*, 198–210.
70. Shahzad, Z.; Gosti, F.; Frérot, H.; Lacombe, E.; Roosens, N.; Saumitou-Laprade, P.; Berthomieu, P., The five AhMTP1 zinc transporters undergo different evolutionary fates towards adaptive evolution to zinc tolerance in Arabidopsis halleri. *Plos Genet.* **2010,** *6*, e1000911.
71. Yuan, L.; Yang, S.; Liu, B.; Zhang, M.; Wu, K., Molecular characterization of a rice metal tolerance protein, *OsMTP1*. *Plant. Cell Rep.* **2012**, *31*, 67–79.
